# Linkage and Association Mapping for Two Major Traits Used in the Maritime Pine Breeding Program: Height Growth and Stem Straightness

**DOI:** 10.1371/journal.pone.0165323

**Published:** 2016-11-02

**Authors:** Jérôme Bartholomé, Marco CAM Bink, Joost van Heerwaarden, Emilie Chancerel, Christophe Boury, Isabelle Lesur, Fikret Isik, Laurent Bouffier, Christophe Plomion

**Affiliations:** 1 BIOGECO, INRA, Univ. Bordeaux, 33610, Cestas, France; 2 Biometris, Wageningen University and Research Centre, NL-6700 AC, Wageningen, Netherlands; 3 HelixVenture, Mérignac, France; 4 North Carolina State University, Department of Forestry and Environmental Resources, Raleigh, NC, United States of America; Nanjing Forestry University, CHINA

## Abstract

**Background:**

Increasing our understanding of the genetic architecture of complex traits, through analyses of genotype-phenotype associations and of the genes/polymorphisms accounting for trait variation, is crucial, to improve the integration of molecular markers into forest tree breeding. In this study, two full-sib families and one breeding population of maritime pine were used to identify quantitative trait loci (QTLs) for height growth and stem straightness, through linkage analysis (LA) and linkage disequilibrium (LD) mapping approaches.

**Results:**

The populations used for LA consisted of two unrelated three-generation full-sib families (*n* = 197 and *n* = 477). These populations were assessed for height growth or stem straightness and genotyped for 248 and 217 markers, respectively. The population used for LD mapping consisted of 661 founders of the first and second generations of the breeding program. This population was phenotyped for the same traits and genotyped for 2,498 single-nucleotide polymorphism (SNP) markers corresponding to 1,652 gene loci. The gene-based reference genetic map of maritime pine was used to localize and compare the QTLs detected by the two approaches, for both traits. LA identified three QTLs for stem straightness and two QTLs for height growth. The LD study yielded seven significant associations (*P* ≤ 0.001): four for stem straightness and three for height growth. No colocalisation was found between QTLs identified by LA and SNPs detected by LD mapping for the same trait.

**Conclusions:**

This study provides the first comparison of LA and LD mapping approaches in maritime pine, highlighting the complementary nature of these two approaches for deciphering the genetic architecture of two mandatory traits of the breeding program.

## Introduction

The genetic variation of key traits used as selection criteria in forest tree breeding programs is estimated by quantitative genetics approaches, in progeny testing and/or common garden experiments [1]. In quantitative genetics, complex traits are considered to be controlled by a large number of independent loci: the so called polygenic model [2, 3]. Quantitative genetics approaches can be used to estimate heritability for the population (i.e. the extent to which phenotypes are controlled by genetic rather than environmental effects), and trait differentiation between populations. A more mechanistic understanding of the genetic architecture of quantitative trait variation, in terms of the number, location, effect and nature of the loci involved, requires analysis of the relationships between DNA polymorphism and phenotypic variation [4]. Quantitative trait loci (QTLs) can be detected by two main approaches: linkage analysis (LA) and linkage disequilibrium (LD) mapping (or genetic association mapping). LA uses information from recombination events between markers within a studied progeny of known pedigree. This approach has been widely used for forest trees since the early 1990s and has led to the detection of QTLs for several traits of economic interest, mostly in biparental crosses [5–7]. LD makes use of historical recombination events in an unknown pedigree from which the study population was derived. This approach has been applied to studies of forest tree genetics over the last decade [8, 9]. It is generally restricted to candidate genes, due to genotyping constraints, and to random mating populations with different levels of population stratification (reviewed in [10]). The resolution of LD mapping is dependent on the level linkage disequilibrium between a DNA marker and a causative variant and therefore on the genome coverage. The detection of an association by LA does not require large numbers of markers, but the mapping resolution obtained with this approach is low, due to the limited number of recombinants. The confidence intervals for QTLs are, therefore, large. By contrast, LD mapping can yield high-resolution maps, particularly for forest tree species in which physical LD decays within one kilobase, at least within the gene space (for a review on conifers see [11]). Genome-wide association studies based on LD mapping therefore require a high marker density for detection of the causal variants, as illustrated by whole-genome resequencing data for poplar [12]. However, for non-model species with large genomes, such as conifers, encouraging results have been obtained with both candidate gene and anonymous marker approaches [10, 13, 14]. Studies combining LD and LA have recently been carried out on forest tree species. This combined approach, taking the best from each approach, has yielded promising results for growth traits in *Populus* hybrids [15] and for adaptive traits in *Picea mariana* [16].

Maritime pine (*Pinus pinaster*) is an important forest tree species in the southwestern part of the Mediterranean basin. It is grown in intensive plantations over large areas of France, Spain and Portugal. The optimization of silvicultural practices and genetic improvements through breeding, selection and testing have had a significant impact on the productivity and quality of plantations established during the last 30 years. A breeding program was first set up for this species in the 1960s, to improve biomass production and stem straightness. The breeding program was initially based on the "Landes" provenance of the Aquitaine region in southern France [17]. Hybrids between the Landes and Corsican provenances have since been introduced into the breeding scheme, to increase genetic gains for stem straightness. Today, 90% of the annual reforestation area (about 20 thousand hectares) is planted with improved seedlings from third-generation seed orchards. Height growth, measured at about 10 years of age, is used as a proxy for wood productivity. The heritability of this trait is generally reported to be low (≈ 0.20) [18–20], but it varies with age and between populations [18, 21]. Stem straightness, measured at the same age, generally displays a higher heritability, of about 0.30 [22], and considerably higher levels of phenotypic variation. The coefficient of variation for stem straightness in maritime pine is about 50–60%, whereas that for height growth is only 10–20% [20]. These two traits display a positive genetic correlation that is unfavorable [20], as the larger trees tend to have less straight (i.e. crooked stem). Knowledge about the genetic architecture of both traits is, therefore, critical, to optimize genetic improvements in one trait without a negative impact on the other trait.

High-density SNP arrays (12 and 9 thousand SNPs) have recently been developed for maritime pine [23, 24]. Given the size of the maritime pine genome (25.8 Gb/C, [25], these arrays cover only a limited proportion of the relevant variation underlying phenotypes. They have nevertheless proved useful for studies of genetic diversity and LD within the Aquitaine breeding population [26], and of the potential of genomic selection for height growth and stem straightness [27]. A series of genetic linkage maps have also been produced on the basis of analyses of the cosegregation of these markers in several maritime pine families. A reference genetic map for maritime pine was recently produced, combining information from independent studies [28]. This reference map is a major achievement as it provides a representation of the maritime pine genome, the sequence of which has yet to be published, due to the difficulties involved in generating highly contiguous sequences for conifer genomes [29–31].

The objective of this study was to determine the genetic architecture of height growth and stem straightness, two major traits of the maritime pine breeding program, by LA and LD mapping approaches. LA was applied to two mapping progenies, one specifically designed to maximize the segregation of height growth, and the other designed to maximize the segregation of stem straightness. The LD approach was applied to a set of trees from breeding populations in which these two traits are used as selection criteria. The QTL results for the LA and LD mapping approaches were compared, to check for consistency and complementarity.

## Materials and Methods

### Plant material, phenotyping and genotyping

#### LA mapping populations

***Three-generation inbred mapping population*–**The first mapping population considered for QTL mapping was a three-generation inbred progeny (F2 population) obtained by the self-pollination of a “Landes x Corsica” hybrid. This accession, called H12, originated from the control cross between genotypes L146 (a female tree from the Landes provenance) and C10 (a male tree from the Corsica provenance). This cross was specially designed for dissection of the genetic architecture of stem straightness (STR). STR is the characteristic differing most markedly between the Landes and Corsican ecotypes of maritime pine: Corsican ecotype being straighter than Landes ecotype [32]. An F2 progeny of a cross between these two ecotypes was therefore ideal for the QTL mapping of this trait (S1 Fig). The trial was established with one-year-old seedlings in March 1999, at Lacanau de Mios, France. The trees were planted in rows with 2 m between trees in the same row and 4 m between rows. STR was estimated as the deviation from the vertical (90° relative to the ground), at breast height, after nine growing seasons, in December 2008. The distribution of the raw data was highly skewed towards low values, with significant deviation from normality in Shapiro-Wilks tests. We therefore used square root-transformed data for QTL detection. Height growth (HT) was also measured at same age. No significant phenotypic correlation was found between these two traits (r = 0.03) contrary to what was found in the Landes provenance with a larger genetic basis [20].

Two medium-throughput genotyping technologies were used to genotype the F2 population: the Goldengate VeraCode (Illumina, San Diego, CA, USA) and MassARRAY iPLEX (Sequenom, San Diego, CA, USA) systems. The design of the SNP arrays is detailed in S1 Note and S2 Fig. Young needles from each tree were harvested and stored at −80°C until DNA extraction, as previously described [33]. All concentrations were determined in fluorescence assays (Quant-IT kit, Invitrogen, Carlsbad, CA, USA). We genotyped 477 individuals with the VeraCode platform and 381 (a subset of the 477) with the iPLEX platform. The genotyping of the F2 population is described in detail in S2 Note. The map of the inter-provenance hybrid tree (H12) was constructed from the data obtained with the two SNP array systems. Mapping was carried out as described by Chancerel *et al*. [33]. JoinMap v4.1 [34] was used to construct the genetic linkage map. Marker order and relative genetic distances were calculated with the regression mapping algorithm and the following parameters: Kosambi mapping function and a LOD threshold ≥ 3. This procedure generated three different maps with different levels of statistical support (map1, map2 and map3, in descending order of statistical support). Map1 was retained for the QTL analysis. Besides, we used χ^2^ tests to test if the allelic segregation of each locus fitted the expected 1:2:1 Mendelian segregation ratio.

***Three-generation outbred mapping population***–The second QTL mapping population was a three-generation outbred progeny (G2 population), specifically designed for elucidation of the genetic architecture of height growth. The four grandparents were selected from the base population (G0 trees). These trees were subjected to progeny testing between 1970 and 1980 and were classified on the basis of breeding value as 'Vigor +' for vigorous trees or 'Vigor–' for less vigorous trees. Two G1 trees obtained from two different biparental 'Vigor +' x 'Vigor–' crosses were then crossed, and the resulting G2 seedlings were planted in autumn 1982, in Malente, France. The seedlings were planted in rows, with 1.1 m between trees within the same row and 4 m between rows. The G2 trees were felled in March 1997, when they were 15 years old, for retrospective height increment analysis. The structure of maritime pine, with a tier of branches at the top of each annual shoot, facilitates retrospective measurement of the length of successive annual shoots along the trunk. We were able to measure 12 annual height increments (between 1985 and 1997) precisely, on 197 trees. STR was not assessed, because this trait did not segregate in this family. Genotyping and linkage analysis were performed in previous studies of this population [25, 35]. Briefly, one genetic map was obtained for each parent, on the basis of the data for 202 G2 trees. In total, 115 AFLP markers were located on the female tree map, and 102 AFLP markers were located on the male tree map.

#### LD mapping population

The population used for LD mapping was described in a previous study by Isik *et al*. [27]. Briefly, the association mapping population consisted of 184 unrelated founders (G0 trees) and 477 individuals from the first generation (G1 trees) of the maritime pine breeding population. These 661 individuals from the Landes provenance (and collateral relatives) have been subjected to progeny testing since the 1960s. Breeding values for HT and STR for the 661 selected trees, at an age of 10 years, were extracted from the maritime pine breeding database and used as pseudo phenotypes (Bouffier L, unpublished). In total, 2,600 SNPs from an Infinium SNP array [23] have already been shown to be informative in this population [27]. We removed five SNPs, for which more than 5% of the data were missing, from the analysis. We also discarded all SNPs with a minor allele frequency (MAF) below 5%, to prevent the identification of spurious marker-trait associations. We therefore used data for 2,498 SNPs (distributed in 1,652 contigs, [36]) for the analysis of marker-trait association. The proportion of missing data was below 2% for all individuals, and no individuals were excluded. The trait-associated SNPs were projected onto the reference map for maritime pine (see below).

### QTL mapping strategy

We used the multiple QTL mapping procedure implemented in the R package *qtl* [37, 38] for the QTL detection on the parental linkage map(s) of each mapping population (F2 and G2). We used the function *stepwiseqtl* with the imputation method and a maximum of five QTLs for forward selection. In total, 1000 permutations were performed for each trait with the function *scantwo*, to estimate the type I error rate at genome level. A threshold error rate of 5% was used to define significant QTLs. The 95% Bayesian credible interval for each QTL was calculated using the function *bayesint* with default parameter [39]. The effects of the QTL as well as the percentage of the explained phenotypic variance were also calculated. For the G2 mapping population the allelic substitution effect of a QTL was calculated as follows: *s* = *μ_AB_* − *μ_AA_*. For the F2 mapping population the additive (a) and dominance (d) effects of a QTL were calculated as follows: *a* = (*μ_BB_* − *μ_AA_*)/2 and *d* = *μ_AB_* − (*μ_BB_* − *μ_AA_*)/2.

### Association mapping strategy

#### Population structure and kinship coefficients

For the LD approach, the putative population structure had already been analyzed by Plomion *et al*. [26] for the 184 G0 trees. They reported an absence of structure in the population consisting of these founders of the breeding program. Known and cryptic relationships between individuals were estimated from pedigree information and SNP data. The additive genetic relationship matrix (**A**) was calculated from pedigree information for the 661 individuals. The 184 G0 trees were considered to be unrelated. A complete pedigree (mother and father known) was available for 355 (74.4%) of the 477 G1 trees, whereas only the mother was known for the remaining trees. In addition to matrix **A**, we also calculated the realized coefficients of relationship (assembled in matrix **G**) between the 661 individuals from the available marker data (2,498 SNPs). Matrix **G** was constructed as follows [40]:

$$G=\frac{(M-P){\left(M-P\right)}^{\prime }}{2\sum p_{i}(1-p_{i})}$$

Where **M** is a matrix of dimensions *n* (number of individuals) × *p* (number of markers) containing the three classes of marker alleles for each individual. One homozygote is coded– 1, the heterozygote is coded 0, and the other homozygote as coded 1. **P** is a matrix of dimensions *n* × *p* containing marker allele frequencies calculated as follows: 2(*p*_*i*_− 0.5), where *p*_*i*_ is the observed allele frequency for marker *i*.

#### Marker-trait association analysis and estimation of marker effects

Associations between SNPs and the two traits (HT and STR) were analyzed with the R package *GenABEL* [41]. In addition to analyses based on a simple model with no structure or kinship effects, two analyses accounting for multiple degrees of relatedness (population stratification and family relationships) were carried out: the family-based score test for association (FASTA, [42]) and the genome-wide rapid analysis using mixed models and regression (GRAMMAR, [43]). The *polygenic* function was used in combination with *mmscore* (FASTA) or *grammar* (GRAMMAR-gamma) in *GenABEL* for the analysis. Given the absence of population stratification [26], only relatedness between individuals was taken into account for the association analysis [44]. For both the FASTA and GRAMMAR models, the **A** and **G** matrices were compared, to determine whether family relationships led to *p*-value inflation. The genomic inflation factor (λ) was calculated [45]. The test-statistic–log_10_(p-value) was used to visualize and identify marker-trait associations exceeding a threshold of 3 (*p* < 0.001), which were considered to be significant. Significance levels were adjusted for multiple testing by the false discovery rate method [46], to obtain q-values with a significance threshold of 0.1.

In addition to classical single marker-trait association studies, we estimated marker effects with two different genomic prediction models: ridge regression best linear unbiased prediction (RR-BLUP,) and Bayesian least absolute shrinkage and selection operator (B-LASSO). The R packages *synbreed* [47] and *BLR* [48] were used to perform RR-BLUP and B-LASSO, respectively. For the B-LASSO model, hyperparameter values were defined as described by Pérez *et al*. [48]. In total, 50,000 iterations were used, with a burn-in of 10,000 runs.

### Projection of LD and LA results onto the reference map of maritime pine

A composite linkage map of maritime pine was established by merging 14 component maps obtained by genotyping seven mapping progenies, including the three-generation inbred (F2) and outbred (G2) mapping populations studied here. This reference map was produced with the R package *LPmerge* [49], by de Miguel *et al*. [28]. For the F2 population, 79% of the 248 mapped SNPs could be localized on the composite map (S1 Table). For the G2 population, the AFLP markers used to construct the female and male parental maps for the G2 population were combined with SNP markers [33] that were also included in the composite map. The shared SNP markers were used to align the three linkage maps with the composite map (S3 Fig). Marker order was highly conserved between the reference map and the parental maps produced from data for the mapping populations studied. Only 1.2%, 6% and 7.6% of the common markers were inverted for the F2 map, the G2 male map and the G2 female map, respectively. Marker inversions occurred only with tightly linked loci (separated by less than 2 cM). This high degree of collinearity between maps made it possible to project the QTLs detected on the F2 and G2 maps onto a single reference map with Biomercator V4.2 [50]. For the association mapping population, 2,392 of the 2,498 SNPs available for marker-trait association were assigned to a genetic map position on the reference map (S4 Fig). This reference map facilitated the direct comparison of QTLs detected by LA and LD mapping. The reference linkage map and the position of the projected QTLs were drawn with MapChart2.2 [51].

## Results

### QTL mapping

#### Genetic linkage map construction for the F2 progeny

In total, 279 (VeraCode) and 76 (iPLEX) polymorphic SNPs were available for construction of the linkage map of the hybrid parent of the F2 population. The final map included 248 markers: 200 VeraCode and 48 iPLEX SNPs. Information for each marker and its location on the genetic maps is provided in S1 Table. The map covered a total of 1,754cM, spread over 12 linkage groups (LGs), corresponding to the haploid number chromosomes of the pine genome. Mean LG length was 146.1 cM (Table 1). The number of markers mapped per LG ranged from 15 (LG1) to 29 (LG3), with a mean value of 20.6, giving a marker density of one marker every 7.7 cM on average. Markers presenting significant segregation distortion (*p* < 0.01) accounted for 3.6% of the mapped markers. These SNPs with segregation distortion were located in four different LGs and represent four different regions (S5 Fig). The highest levels of distortion were found on LG8 and LG2. The region of LG2 was already highlighted in a previous study on the same cross [23].

**Table 1:** Descriptive statistics for the genetic linkage map of the parental genotype (H12 hybrid) of the F2 mapping population.

Linkage group	Length (cM)	Number of SNPs	Mean inter-marker interval (cM)	Number of distorted SNPs (%)
1	146.7	15	10.5	0
2	135.3	17	8.5	3 (17.6%)
3	159.7	29	5.7	0
4	165.4	20	8.7	2 (10%)
5	169.7	23	7.7	0
6	121.3	26	4.9	0
7	148.6	23	6.8	0
8	157.9	18	9.3	2 (11.1%)
9	136.7	19	7.6	1 (5.3%)
10	161.9	18	9.5	0
11	118.2	17	7.4	0
12	132.6	23	6	1 (4.3%)
Total	1,754	248	7.7	9 (3.6%)

#### QTL detection by LA in the F2 and G2 progenies

**Fig 1:**
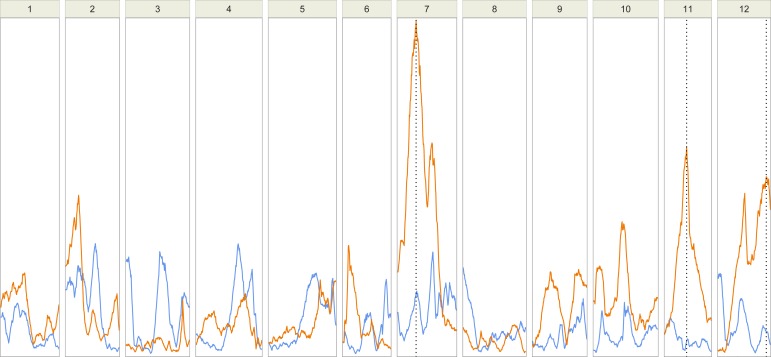
Results from QTL analysis of the F2 mapping population. The LOD score patterns for total height (blue) and stem straightness (orange) over the 12 linkage groups of maritime pine are represented. The location of the QTLs (p < 0.05 at the genome wide level) is indicated by vertical dotted lines.

***F2 population***–As expected from previous studies, stem straightness had a high coefficient of variation (CVp = 0.68, S2 Table) probably exacerbated by the type of cross used: a self-progeny from a hybrid between trees of the Landes and Corsica provenances. No correlation was found between STR and HT measured at the same age. Three QTLs located on LG7, 11 and 12 were detected for STR and no QTL was detected for HT (Table 2, Fig 1). The percentage of phenotypic variance explained by each of these QTLs was relatively small and ranged from 3.19% to 5.47%. All together the QTLs for STR explained 13.16% of the phenotypic variance. The favorable allele for QTLs related to STR came from the Corsican grandparent on LG11 and LG12, and from the Landes grandparent on LG7 (S6 Fig).

**Table 2:** QTL results from the analysis of the F2 mapping population for stem straightness (STR). Location (cM) of QTLs on linkage groups with 95% Bayesian credible intervals, % variance explained by the QTL (PEV), log10 likelihood ratio scores (LOD) and grandparental origin of the favorable effect (Landes (L) or Corsican (C)) are presented. For the additive and dominance effects, the proportions in standard deviation is indicated in parenthesis.

Trait	Linkage group	Position (cM)	LOD score	95% Bayesian credible interval (cM)	PEV	PEV tot	Additive effect	Favorable allele	Dominance effect
STR	7	47	6.49	35–57	5.47	13.16	0.53 (0.34σ)	L	0.194 (0.12 σ)
STR	11	56	4.05	38–64	3.56		0.42 (0.27 σ)	C	-0.003 (0 σ)
STR	12	122	3.47	56–132.57	3.19		0.34 (0.22 σ)	C	-0.273 (0.17 σ)

***G2 population***–The coefficient of phenotypic variation for height increments ranged from 0.17 to 0.44, with a trend towards a decrease over time (S2 Table). The coefficients of correlation between height increments ranged from 0.2 to 0.71 and were higher for height increments in years that were close together (S3 Table). QTL analyses led to the detection of one QTL on the male map and one QTL on the female map (Table 3). For the male parent, the percentage of the phenotypic variance explained by a QTL was 3.54%. For the female parent, the QTL identified for height increments accounted for up to 4.17% of the observed phenotypic variance.

**Table 3:** QTL results for the analysis of the G2 mapping population for height increment. Location (cM) of QTLs on linkage groups with 95% Bayesian credible intervals, % variance explained by the QTL (PEV) and log10 likelihood ratio scores (LOD) are presented. For the allelic substitution effect, the proportion of standard deviation is indicated in parenthesis.

Parental map	Trait	Linkage group	Position (cM)	LOD score	Bayesian credible interval (cM)	PEV	Allelic substitution effect
Male	HI96	7	0	2.39	0–43	3.54	6.2 (0.4 σ)
Female	HI87	1	28.15	2.69	12.15–55.11	4.17	9.43 (0.43 σ)

### Association mapping

#### Relatedness between genotypes

The comparison between the expected kinship coefficients obtained with pedigree information (matrix **A**) and the realized kinship coefficients obtained with genomic information (matrix **G**) highlighted differences between the two estimates (S7 Fig). Indeed, marker-based analysis revealed inconsistencies in the pedigree for 39 G1 and nine G0 trees. Kinship coefficients (between G0 and G1 and within G1) based on pedigree data were therefore considered erroneous for these individuals (e.g. 0 instead of 0.5 for the parent-offspring relationship). The 39 G1 and nine G0 individuals presenting inconsistencies between pedigree-based and marker-based kinship findings were removed for subsequent analyses. Marker data also identified the male parents of eight G1 individuals. As reported by Plomion *et al*. [26], genomic relatedness between the G0 individuals was weak or absent (S7 Fig).

#### Marker-trait association

**Fig 2:**
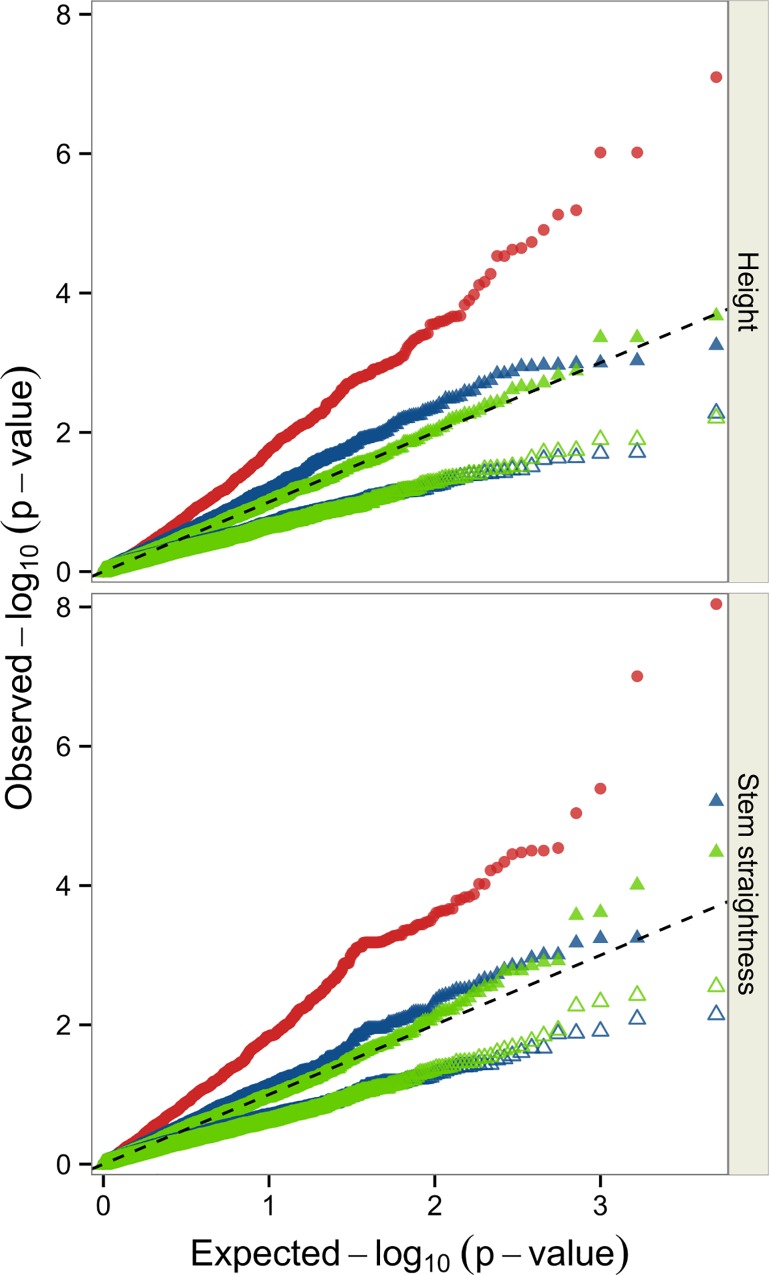
Q-Q plots for the three different association models. Simple model (red dots), FASTA model (closed triangles) and Grammar-GAMMA model (open triangles). For both the FASTA and Grammar-GAMMA models, we used matrix **A** (in blue) or matrix **G** (in green) to take relatedness between individuals into account.

Relatedness between individuals is known to bias *p*-values in association analysis. We therefore compared different models, using representations of the observed and expected–log_10_(p-value) values on Q-Q plots (Fig 2). As expected, the model excluding kinship effects displayed higher *p*-value inflation, with λ = 1.84 for HT and λ = 2.49 for STR. Smaller inflation factors were obtained for the FASTA method including kinship coefficients from matrix **A**: λ = 1.21 for both HT and STR. Almost no bias was observed for the FASTA method including kinship coefficients from matrix **G**: λ = 0.97 for HT and λ = 0.96 for STR. By contrast, deflation was observed for the GRAMMAR-gamma method (Fig 2), with a smaller departure from expectations for a model including kinship coefficients from matrix **A** (λ = 0.53 for HT and λ = 0.60 for STR) than for a model including kinship coefficients from matrix **G** (λ = 0.5 for HT and λ = 0.51 for STR). Given the low level of bias observed with the FASTA method and matrix **G**, we can conclude that population structure was effectively controlled by the family relatedness captured in matrix **G**. We therefore used this model for the detection of marker-trait associations.

**Fig 3:**
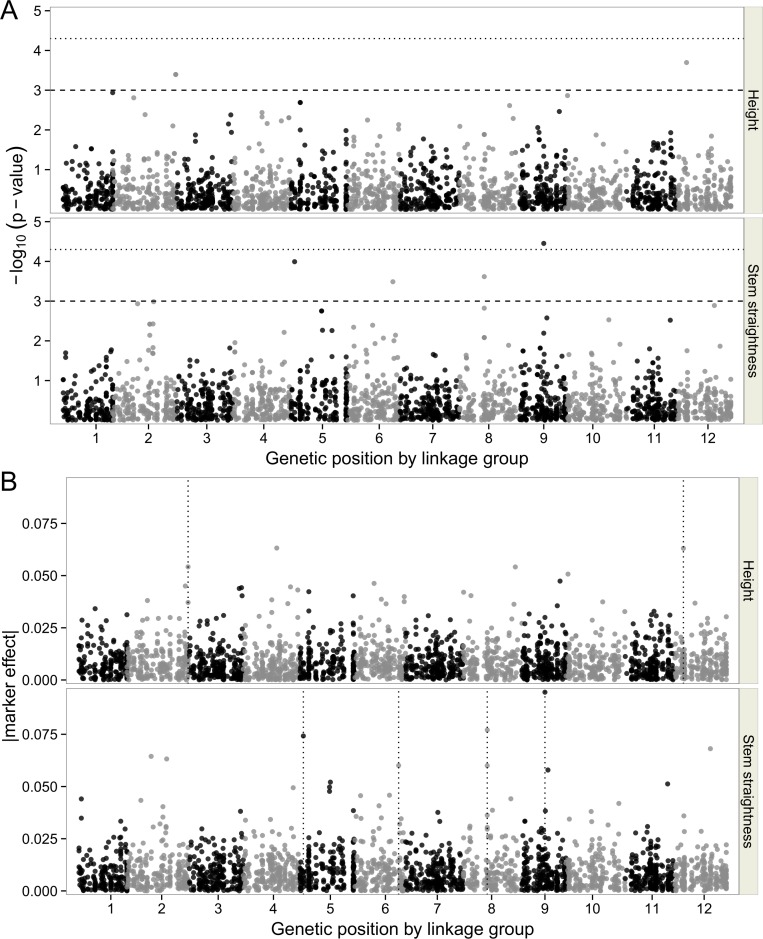
**Distribution of *p-*values (on a negative log**_**10**_ **scale) over the 12 linkage groups from the genome-wide association analysis (panel A) and the absolute effect of markers in the Bayesian LASSO model (panel B) for height and stem straightness.** In panel A, the horizontal dashed line represents the threshold at *p* = 0.001 and the horizontal dotted line represents the threshold at *q* = 0.10. In panel B, the associations for which *p* <0.001 are indicated by vertical dotted lines. Only mapped markers are displayed.

**Fig 4:**
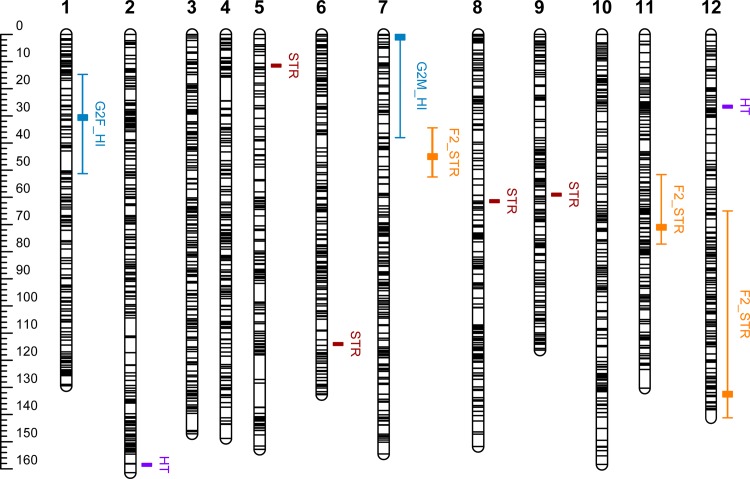
Position of the QTLs detected by LA and LD mapping on the *Pinus pinaster* composite genetic map. For LA mapping, QTLs for stem straightness (STR) are shown in orange and QTLs for height increment (HI) are shown in blue. The whiskers indicate the 95% credible interval around QTL peaks. The locations of markers significantly associated with height (HT, purple) and stem straightness (STR, red) are also indicated. On linkage group 2, the two significant associations for HT are co-located (158 cM and 158.1 cM).

Seven of the 4,996 association tests performed (2,498 SNPs on two traits) yielded significant results (*p*<0.001, Table 4). The *p*-value profiles (in terms of–log_10_(*p*)) for all tested SNPs for both traits were plotted on Manhattan plots (Fig 3A). HT was found to be significantly associated with three SNPs located on three different contigs. Two of these SNPs were located on LG2 (sp_v3.0_unigene209724, sp_v3.0_unigene29702) and one was located on LG12 (sp_v3.0_unigene128161). Four SNPs significantly associated with STR were identified, on LG5 (sp_v3.0_unigene17681), LG6 (sp_v3.0_unigene16979), LG8 (sp_v3.0_unigene31740) and LG9 (sp_v3.0_unigene11934). All the significant SNPs were represented on the reference map of *P*. *pinaster* (Fig 4). After correction for multiple testing according to the FDR method, only one significant SNP for STR remained, on LG9. The putative protein associated with this SNP (accounting for 4.2% of the variation) was a RING finger-like protein (Table 4). Estimates of the effect of SNPs on traits obtained by RR-BLUP and B-LASSO regression were plotted with Manhattan plots (Fig 3B for B-LASSO and S8 Fig for RR-BLUP). The effects of the SNPs associated with HT ranged from -0.029 to 0.035 for RR-BLUP and from -0.044 to 0.078 for B-LASSO (Table 4). Similarly, for STR, the estimates obtained with B-LASSO regression were higher (from -0.087 to 0.080) than those obtained with RR-BLUP (from -0.043 to 0.035), as expected given the different assumptions concerning marker effects between the two regression models. Overall, no coincidence between marker-trait association and QTL positions was found (Fig 4).

**Table 4:** Significant (*p*<0.001) marker-trait pairs and their location on the genetic map, for height (HT) and stem straightness (STR).

Trait	SNP ID	Contig ID ^a^	LG	Position (cM) ^b^	Major allele	Minor allele	Minor allele freq.	*p*-value	Marker effect		Putative function ^c^
									RR-BLUP	B-LASSO	
HT	FN694040-568	sp_v3.0_unigene209724	2	158	T	C	0.26	3.95E-04	-0.029	-0.038	DEAD-box ATP-dependent RNA helicase 13
	FN694040-268	sp_v3.0_unigene29702	2	158.1	C	T	0.26	3.95E-04	-0.029	-0.044	-
	F51TW9001DJ7E6-423	sp_v3.0_unigene128161	12	27.1	C	A	0.40	2.18E-04	0.035	0.078	-
STR	BX249988-154	sp_v3.0_unigene17681	5	12	C	A	0.20	9.01E-05	-0.038	-0.068	Eugenol synthase 1
	AL750210-452	sp_v3.0_unigene16979	6	114.5	T	C	0.16	2.63E-04	0.033	0.067	Homeobox-leucine zipper-like protein
	FN695400-1167	sp_v3.0_unigene31740	8	61.9	G	A	0.19	2.08E-04	0.035	0.080	Abhydrolase domain-containing protein 13
	AL750418-263	sp_v3.0_unigene11934	9	59.5	G	C	0.36	3.38E-05	-0.043	-0.087	RING finger-like protein

^a^ Contig ID from Canales *et al*. [36]

^b^ Position from de Miguel *et al*., [28]

^c^ Annotation from Canales *et al*. [36]

## Discussion

Most traits of interest in forest tree breeding, including height growth and wood properties, are quantitative traits with complex genetic architectures and low to medium heritabilities [52]. The identification of relevant markers from LA and LD studies would therefore improve the prediction of breeding values for individuals from genotypic data alone, thereby increasing the efficiency of selection strategies [53–55]. In this study, we investigated the genetic architecture of height growth and stem straightness, two major traits in the maritime pine breeding program, through a combination of linkage and genome-wide association mapping.

LA identified five regions of the *P*. *pinaster* genome as associated with the traits considered: three regions for stem straightness and two for annual height growth increment. Interestingly, the favorable allele of one of the three detected QTLs for stem straightness comes from the Landes grand-parent. Consistent with this observation made at the molecular level, it should be mentioned that stem straightness is genetically variable within the Landes provenance and heritable, resulting in positive genetic gains for the first breeding generations [20]. The percentage of the variance explained by individual QTLs was small (up to 5.47%) and similar to or lower than that reported for growth traits in previously published studies on conifers [16, 56–60]. For example, Devey *et al*. [60] reported percentages of variance explained of 0.4% to 2.04% for diameter, for a large progeny of *P*. *radiata* including about 500 genotypes. Conversely, in *Pseudotsuga* menziesii, two QTLs were detected for height growth, explaining 16.1 and 17.7% of the phenotypic variation in multiple small (*n* = 10) full-sib families [56]. Various factors, including population size, can lead to an overestimation of the effect of QTLs [61, 62]. Given the experimental design used here (sample size of less than 500 genotypes and no clonal replicates) only strong QTL effects would be detectable. In maritime pine, QTL mapping, based on two- or three-generation pedigrees, has been carried out for height and radial growth [63], water-use efficiency [64, 65], wood properties [35], and traits relating to photosynthesis [66], but no previous study has addressed the genetic architecture of stem straightness. To the best of our knowledge, only one study in a *P*. *elliottii* × *P*. *caribaea* hybrid has addressed the genetic architecture of stem straightness in forest trees [67]. However, this study identified no QTLs for this trait. We identified no QTLs common to STR and HT, which suggests that these two traits have different genetic architectures. Confirmation in other genetic backgrounds is required, but this result is encouraging, as it suggests that it may be possible to overcome the small, but significant negative genetic correlation between these two traits [20].

In addition to QTL mapping in dedicated full-sib families, we also performed genome-wide association with related genotypes from the first two generations of the maritime pine breeding population. Population stratification or relatedness can result in the detection of spurious marker-trait associations [68, 69]. However, no structure was detected in the founder population (G0 trees) [26], so only relatedness between individuals was taken into account, by integrating the realized genomic relationship matrix into the marker-trait association model. This consideration of relatedness considerably reduced *p*-value inflation, for both traits. Similar results were reported for wood quality and growth traits in *Eucalyptus globulus* [70, 71] and for wood property traits in *Cryptomeria japonica* [72]. Only seven of the 2,498 SNPs, from six different loci, were found to be significantly associated with the traits considered (p < 0.001). After correction for multiple testing, only one SNP on LG9, in a gene encoding a RING finger-like protein, remained significantly associated (*Q* < 0.1) with stem straightness. In previous studies of growth traits in conifer, a small number of associations were highlighted through the use of candidate genes [14, 16, 73] or a larger set of markers [13, 74]. In maritime pine, Lepoittevin *et al*. [14] tested 184 SNPs for association with height growth in eight-year-old trees. They identified only one association. In the same species, Cabezas *et al*. [73] identified four SNPs within a single gene (korrigan) as associated with total height and polycyclism in three-year-old maritime pines. LD decays rapidly in maritime pine [27, 75, 76], as in other conifers [77–79]. It has been observed that not only LD decay over a distance less than the size of a single gene, but even two SNPs that are immediately adjacent might be in complete linkage equilibrium, which may reflect that the respective mutations occurred at different places in the coalescent history of the sampled sequences. The few association mapping studies carried out to date have thus considered polymorphisms within carefully selected candidate genes [14, 73, 80]. These studies have yielded promising results with hundreds (as opposed to thousands) of SNP markers. However, none of the genes associated with growth traits in these studies were identified here. In *P*. *taeda*, Cumbie *et al*. [13] found only one SNP associated with height growth among the 3,938 SNPs from as many genes tested. A higher proportion of significant associations was reported by Prunier *et al*. [16], with 20 SNPs from a set of 52 SNPs specifically selected from previous QTL and association mapping studies found to be associated with height. In general, the proportion of marker-trait associations detected for other quantitative traits, such as wood properties [72, 81, 82], adaptive traits [83–85], and metabolite content [86], was slightly higher than that for growth traits. The strategy used (candidate gene-based vs. non-targeted approaches) and the genetic architecture of the traits therefore play a major role in the discovery of marker-trait associations.

As pointed out by Grattapaglia *et al*. [52], tree growth probably involves the interaction of many genetic and epigenetic factors responding dynamically to internal and environmental signals. Low repeatability across environments and populations has thus been reported for QTLs identified by LA or LD mapping [10, 52, 87]. Indeed, marker-trait associations have been difficult to validate. In a study on *P*. *radiata* [82], only two of 10 SNPs associated with wood quality traits in the discovery population (provenance-progeny trial) were also associated with these traits in the validation populations (half-sib families). Moreover, the authors found discrepancies in allele effects between the discovery and validation populations for one SNP, which they suggested might be due to genotype-by-environment interactions. This low repeatability, together with the small proportion of the gene space explored here (in term of both the number of genes sampled and SNPs per gene) as well as the partially different genetic background used (Landes and Corsican ecotypes), might account for the discrepancies between the LA and LD mapping results. Two previous studies that also combined LA and LD mapping approaches to decipher the genetic architecture of growth in black spruce [16] and in poplar [15] reported better consistency between the locations of the QTLs identified by the two approaches. However, unlike this study, they used a two-step strategy in which the QTL regions detected in the first step were used to target specific genomic regions and to select SNPs. Marker-trait association mapping was then performed with the selected SNPs in a population with a broader genetic background.

## Conclusion and Prospects

Two different strategies have been used for LD mapping in conifers: i) early studies focused on a selected set of candidate genes with the depth of SNP coverage clearly favored, resulting in the discovery of significant associations [16, 73, 82, 88], ii) later studies, including this one, have made use of higher-throughput genotyping platforms, resulting in a greater emphasis on the breadth of SNP coverage, *i*.*e*. a larger number of genes but a smaller number of SNPs per gene [13, 72, 86]. This approach has generally identified smaller numbers of associations, due to the low physical LD between the causal polymorphisms of markers in these outbreeding species with large effective population sizes.

Current technologies have made it possible to capture and sequence the coding fraction of any conifer genome [89]. Such approaches should facilitate the discovery of causal variants within the coding sequences of genes, but it remains unclear whether increasing both marker density and the sample size of the discovery population will make it possible to account for a large proportion of the phenotypic variance of targeted traits, as shown for height in humans [90]. Sequence capture should also allow targeting the regulatory fraction of the genome, but this exploration will require a better contiguity at least within the gene space that is currently available (S6 Table). Besides, the next decade will probably see a shift from gene-to-gene to gene network approaches, with the accumulation of functional information, as well as the consideration of epigenetic mechanisms [91]. For instance, a loss of stem straightness is associated with hormone regulation in the vascular cambium and secondary wood-forming tissue [92]. A deviation from verticality results in the formation of compression wood on the lower side of the leaning stem, which tends to restore the vertical position of the stem. The advances in high-throughput molecular technologies made over the last 15 years have led to improvements in our understanding of the interactions between hormones, transcription factors and other regulatory molecules, such as microRNAs, in secondary growth and wood formation (reviewed in [93]). Integrating knowledge about the regulatory network of interacting genes into genome-wide association studies should improve our understanding of genotype-phenotype maps [94].

## Supporting Information

S1 Data Datasets for the F2 mapping population and the LD mapping population. (XLSX)

S1 Fig Segregation for stem straightness (STR) in the F2 mapping population. (PDF)

S2 Fig Flowchart describing the different steps used to identify putative SNPs in the hybrid parent (H12) of the F2 progeny. (PDF)

S3 Fig **Comparison between the composite map of *P*. *pinaster* and the F2 map (panel A), G2 male map (panel B) and G2 female map (panel C).** The composite map is represented in blue and the parental maps of the F2 and G2 populations in green. The numbers at the top of each linkage group indicate the number of markers common to different maps for each linkage group (LG).
(PDF)

S4 Fig Genetic location of the markers used for association mapping on the composite linkage map of *Pinus pinaster* established by de Miguel *et al*. (2015). The number of markers per linkage group is indicated beneath each linkage group.
(PDF)

S5 Fig Distribution of the p-value of χ^2^ tests for the goodness-of-fit to the expected Mendelian segregation ratios of along the 12 linkage groups of F2 genetic map. Horizontal dotted lines represent the threshold above which SNPs are significantly distorted (p < 0.01).
(PDF)

S6 Fig Average stem straightness (estimated as the deviation from verticality) as a function of genotype at three QTL loci on chromosomes 7 (sp_v3.0_unigene17779), 11 (sp_v3.0_unigene175515) and 12 (sp_v3.0_unigene20418). The genotype of the grandparents (Landes or Corsican) is indicated below the corresponding class.
(PDF)

S7 Fig Scatterplot of additive relationship coefficients and genomic relationship coefficients within generations (G0 or G1) and between generations (G0-G1). (PDF)

S8 Fig Single marker effects on total height and stem straightness, in ridge regression BLUP (RR-BLUP). The absolute values of markers are plotted on the 12 linkage groups of the *Pinus pinaster* composite map.
(PDF)

S1 Note Design of the SNP arrays for the genotyping of the F2 mapping population. (PDF)

S2 Note Genotyping of the F2 mapping population. (PDF)

S1 Table List of the 248 mapped SNPs for the F2 mapping population. The following information is presented: the contig and SNP IDs, the associated dbSNP ss accession number, the genotyping platform used, the alleles considered, the designability score from Illumina (ADT software), the associated linkage group, and the position on the F2 map and on the *Pinus pinaster* composite map when available. (see separate supporting information file).
(XLSX)

S2 Table Descriptive statistics for the traits measured in the F2 and G2 mapping populations. For the F2 mapping population: stem straightness (STR) and height growth (HT) and for the G2 mapping population: annual height increment (HI) from 1985 to 1997.
(PDF)

S3 Table Spearman correlations for height increment in the G2 mapping population. Only significant correlations (*p* < 0.05) are listed.
(PDF)

S4 Table Statistics for the two VeraCode SNP arrays. (PDF)

S5 Table Information obtained from MassArray assay design software (see separate supporting information file). (XLSX)

S6 Table Current status of major international conifer genome sequencing projects. (PDF)
