# Correction: Linkage and Association Mapping for Two Major Traits Used in the Maritime Pine Breeding Program: Height Growth and Stem Straightness

**DOI:** 10.1371/journal.pone.0171439

**Published:** 2017-01-30

**Authors:** Jérôme Bartholomé, Marco CAM Bink, Joost van Heerwaarden, Emilie Chancerel, Christophe Boury, Isabelle Lesur, Fikret Isik, Laurent Bouffier, Christophe Plomion

There are missing axes in Fig 1. Please view the correct Fig 1 here.

**Fig 1:**
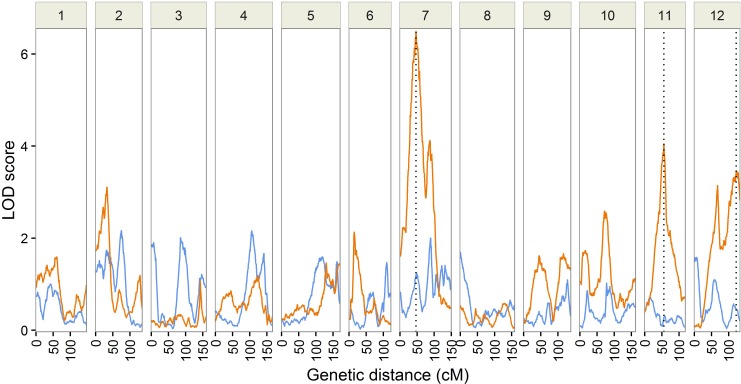
Results from QTL analysis of the F2 mapping population. The LOD score patterns for total height (blue) and stem straightness (orange) over the 12 linkage groups of maritime pine are represented. The location of the QTLs (p < 0.05 at the genome wide level) is indicated by vertical dotted lines.
