# Supplementary figures and images for: Linkage and Association Mapping for Two Major Traits Used in the Maritime Pine Breeding Program: Height Growth and Stem Straightness
Source: PLoS One. 2016 Nov 2;11(11):e0165323. doi: 10.1371/journal.pone.0165323 (PMC5091878; doi:10.1371/journal.pone.0165323)

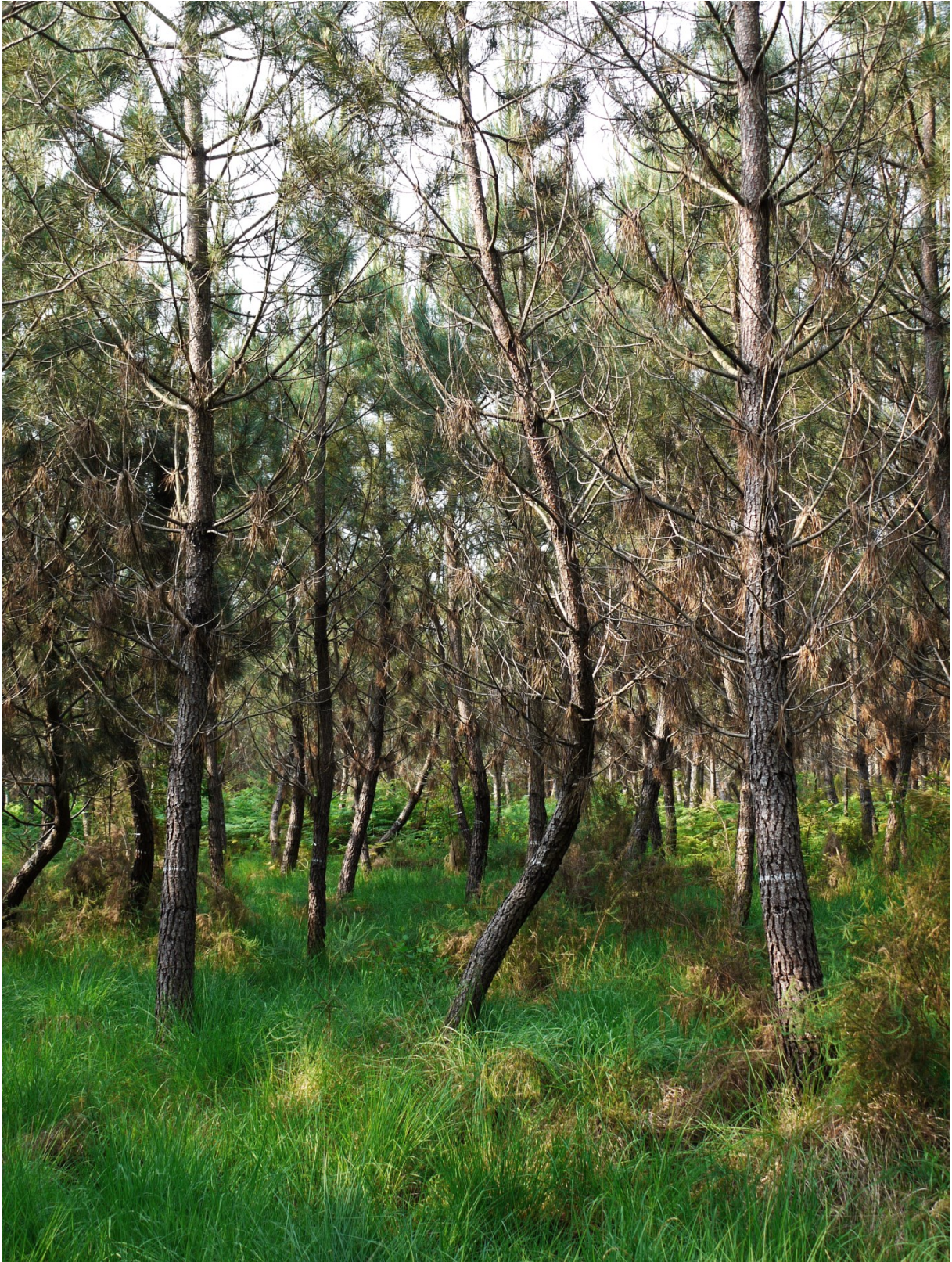

**S1 Fig. Segregation for stem straightness in the F2 mapping population**

Supplement: S1 Fig — (PDF) [file pone.0165323.s002.pdf]
