# Supplementary material for: Linkage and Association Mapping for Two Major Traits Used in the Maritime Pine Breeding Program: Height Growth and Stem Straightness
Source: PLoS One. 2016 Nov 2;11(11):e0165323. doi: 10.1371/journal.pone.0165323 (PMC5091878; doi:10.1371/journal.pone.0165323)
Supplement: S2 Fig — (PDF) [file pone.0165323.s003.pdf]

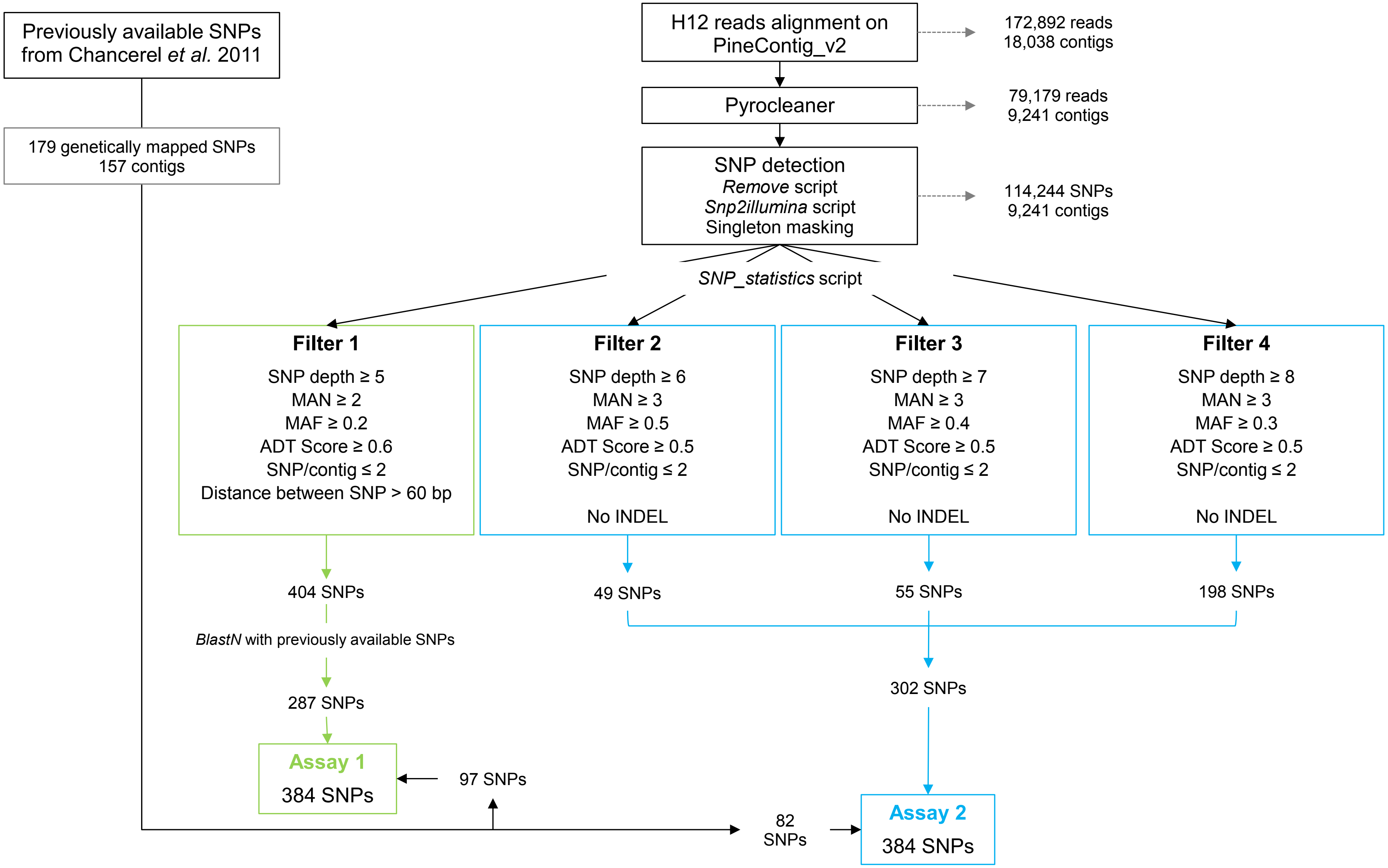

S2 Fig. Flowchart describing the different steps used to identify putative SNPs in the hybrid parent (H12) of the F2 progeny.
