# Supplementary material for: Linkage and Association Mapping for Two Major Traits Used in the Maritime Pine Breeding Program: Height Growth and Stem Straightness
Source: PLoS One. 2016 Nov 2;11(11):e0165323. doi: 10.1371/journal.pone.0165323 (PMC5091878; doi:10.1371/journal.pone.0165323)
Supplement: S3 Fig — Comparison between the composite map of P. pinaster and the F2 map (panel A), G2 male map (panel B) and G2 female map (panel C). The composite map is represented in blue and the parental maps of the F2 and G2 populations in green. The numbers at the top of each linkage group indicate the number of markers common to different maps for each linkage group (LG). (PDF) [file pone.0165323.s004.pdf]

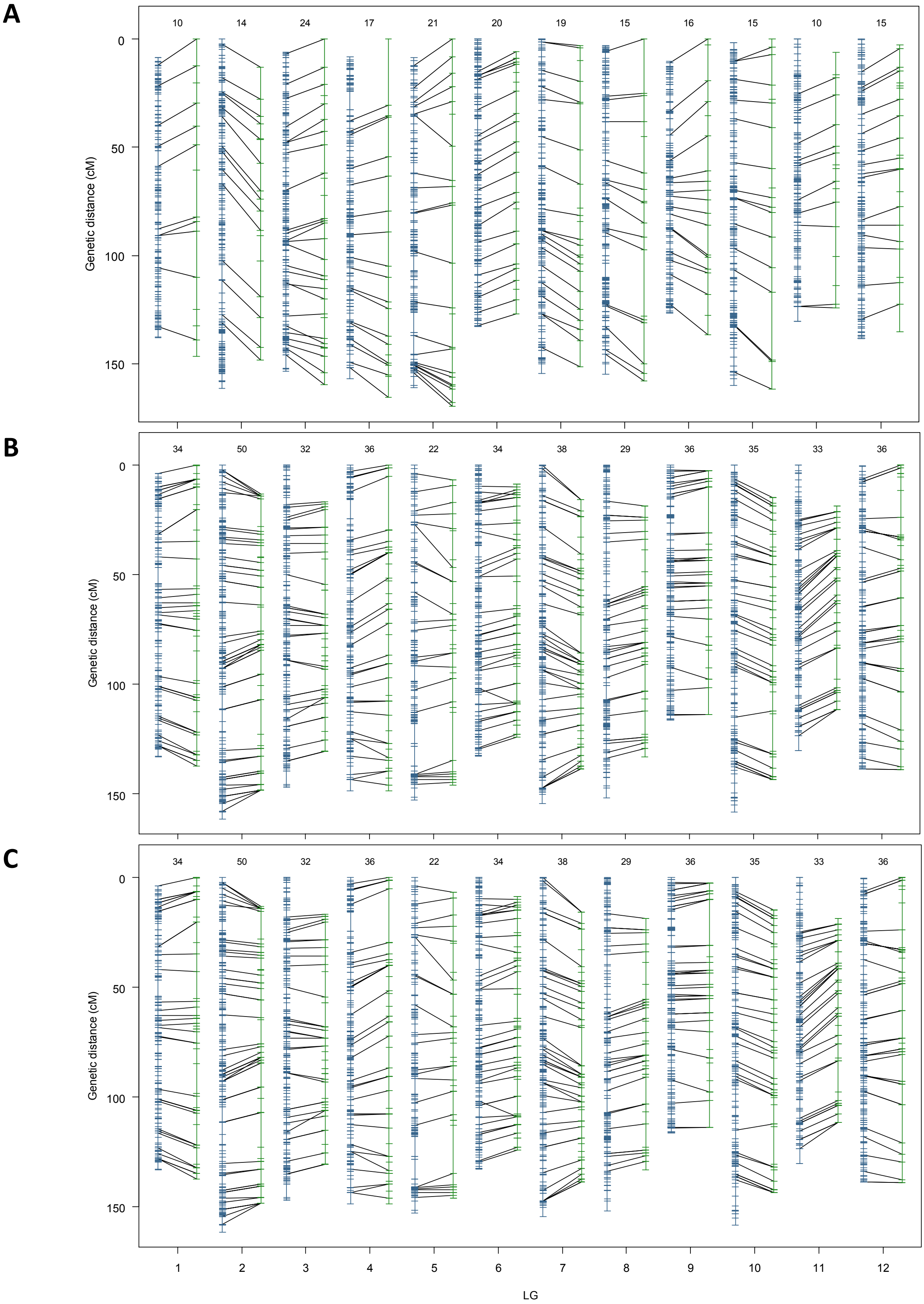

S3 Fig. Comparison between the composite map of *P. pinaster* and the F2 map (panel A), G2 male map (panel B) and G2 female map (panel C). The composite map is represented in blue and the parental maps of the F2 and G2 populations in green. The numbers at the top of each linkage group indicate the number of markers common to different maps for each linkage group (LG).
