# Supplementary material for: Linkage and Association Mapping for Two Major Traits Used in the Maritime Pine Breeding Program: Height Growth and Stem Straightness
Source: PLoS One. 2016 Nov 2;11(11):e0165323. doi: 10.1371/journal.pone.0165323 (PMC5091878; doi:10.1371/journal.pone.0165323)
Supplement: S4 Fig — The number of markers per linkage group is indicated beneath each linkage group. (PDF) [file pone.0165323.s005.pdf]

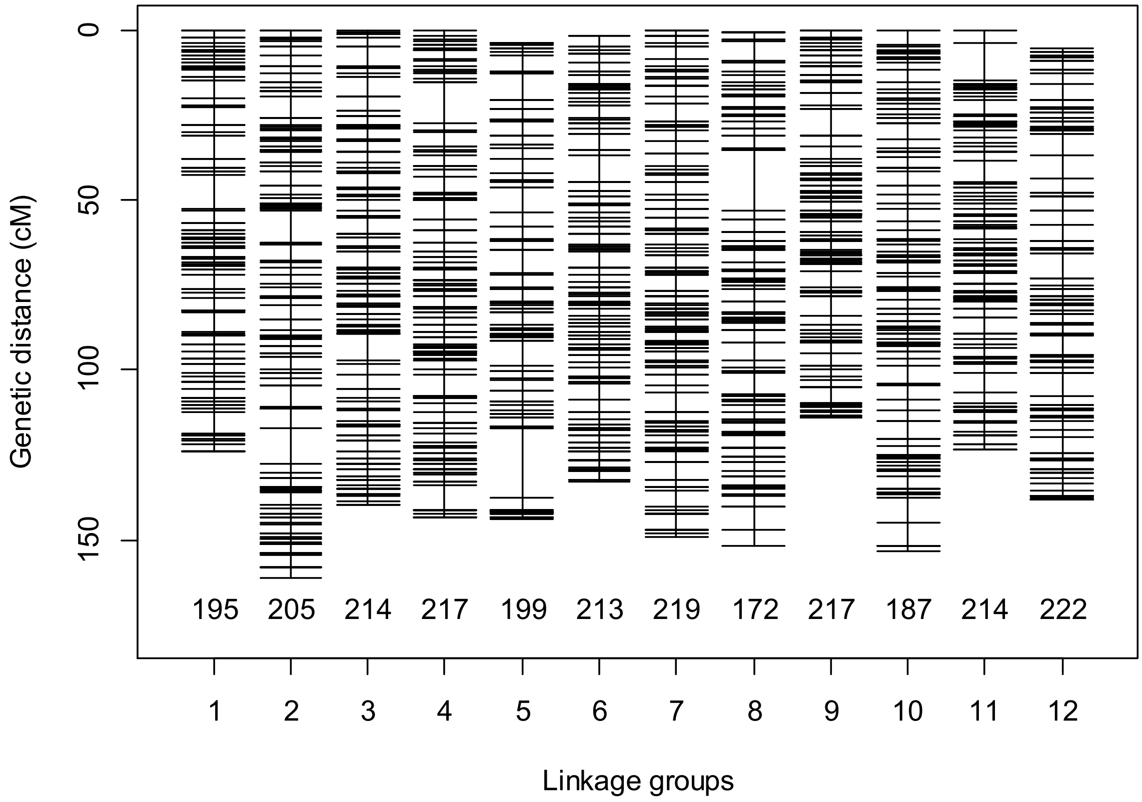

S4 Fig. Genetic location of the markers used for association mapping on the composite linkage map of *Pinus pinaster* established by de Miguel *et al.* (2015). The number of markers per linkage group is indicated beneath each linkage group.
