# Supplementary material for: Linkage and Association Mapping for Two Major Traits Used in the Maritime Pine Breeding Program: Height Growth and Stem Straightness
Source: PLoS One. 2016 Nov 2;11(11):e0165323. doi: 10.1371/journal.pone.0165323 (PMC5091878; doi:10.1371/journal.pone.0165323)
Supplement: S5 Fig — Horizontal dotted lines represent the threshold above which SNPs are significantly distorted (p < 0.01). (PDF) [file pone.0165323.s006.pdf]

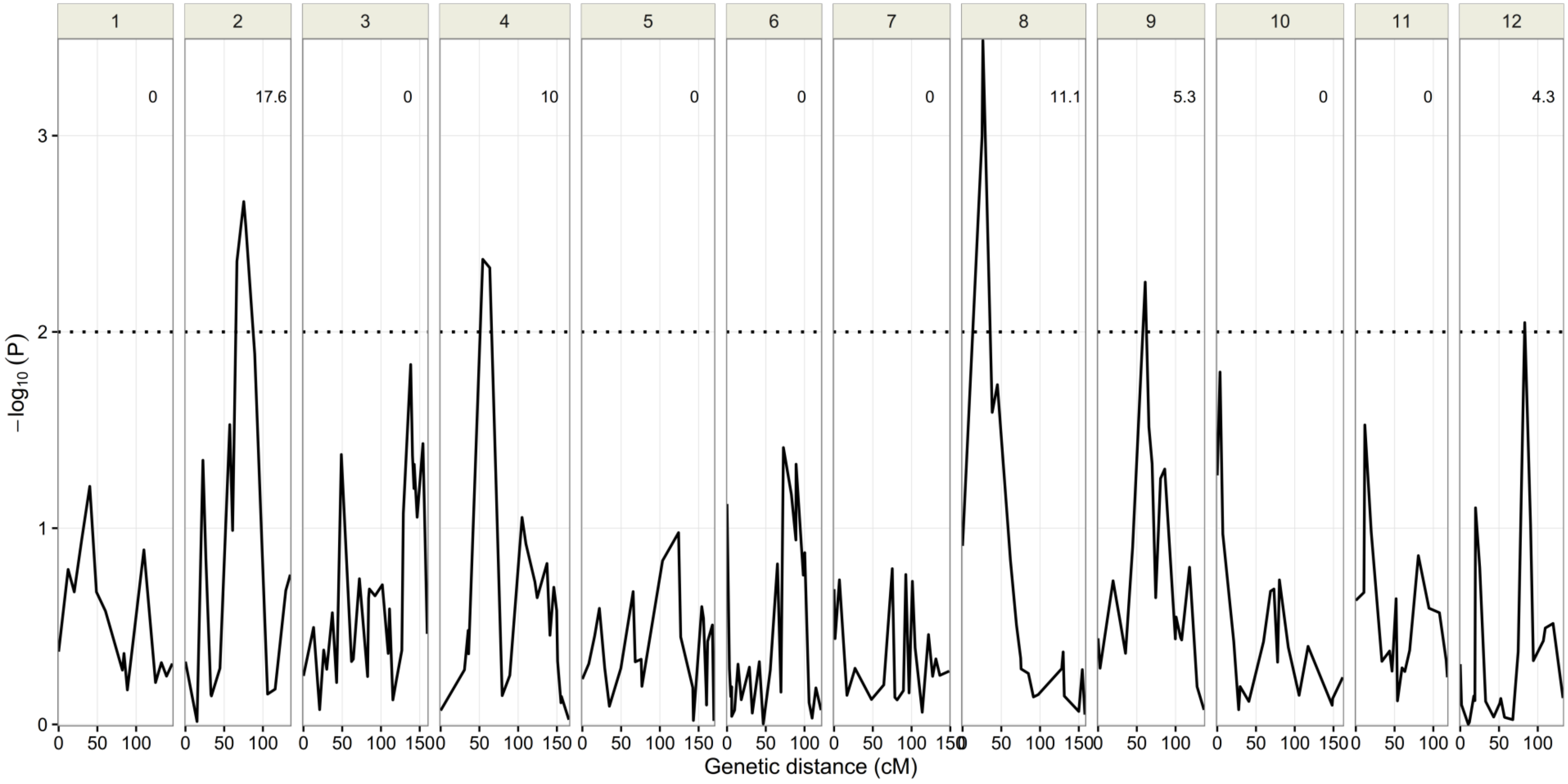

S5 Fig. Distribution of the p-value of χ² tests for the goodness-of-fit to the expected Mendelian segregation ratios of along the 12 linkage groups of F2 genetic map. Horizontal dotted lines represent the threshold above which SNPs are significantly distorted ($p < 0.01$).
