# Supplementary material for: Linkage and Association Mapping for Two Major Traits Used in the Maritime Pine Breeding Program: Height Growth and Stem Straightness
Source: PLoS One. 2016 Nov 2;11(11):e0165323. doi: 10.1371/journal.pone.0165323 (PMC5091878; doi:10.1371/journal.pone.0165323)
Supplement: S6 Fig — The genotype of the grandparents (Landes or Corsican) is indicated below the corresponding class. (PDF) [file pone.0165323.s007.pdf]

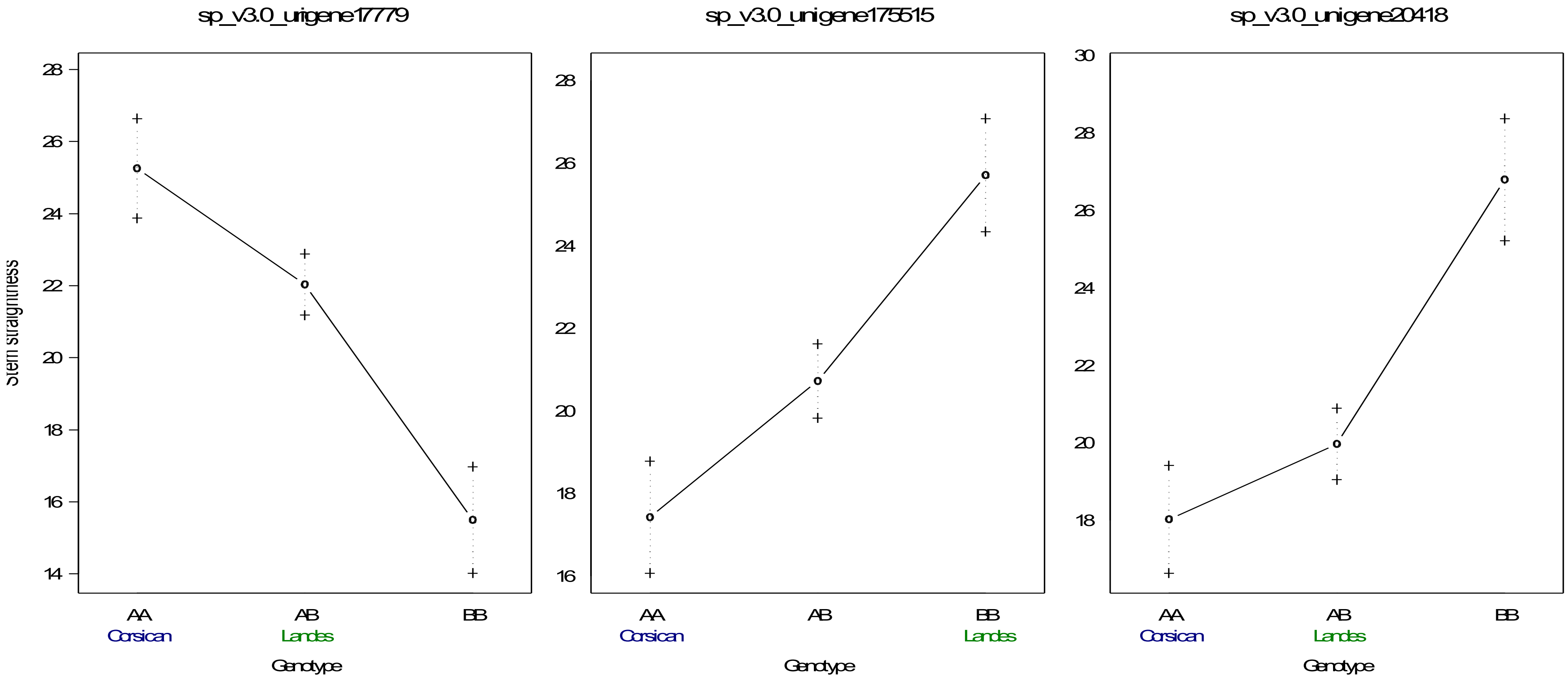

S6 Fig. Average stem straightness (estimated as the deviation from verticality) as a function of genotype at three QTL loci on chromosomes 7 (sp_v3.0_unigene17779), 11 (sp_v3.0_unigene175515) and 12 (sp_v3.0_unigene20418). The genotype of the grandparents (Landes or Corsican) is indicated below the corresponding class**.**
