# Supplementary material for: Linkage and Association Mapping for Two Major Traits Used in the Maritime Pine Breeding Program: Height Growth and Stem Straightness
Source: PLoS One. 2016 Nov 2;11(11):e0165323. doi: 10.1371/journal.pone.0165323 (PMC5091878; doi:10.1371/journal.pone.0165323)
Supplement: S7 Fig — (PDF) [file pone.0165323.s008.pdf]

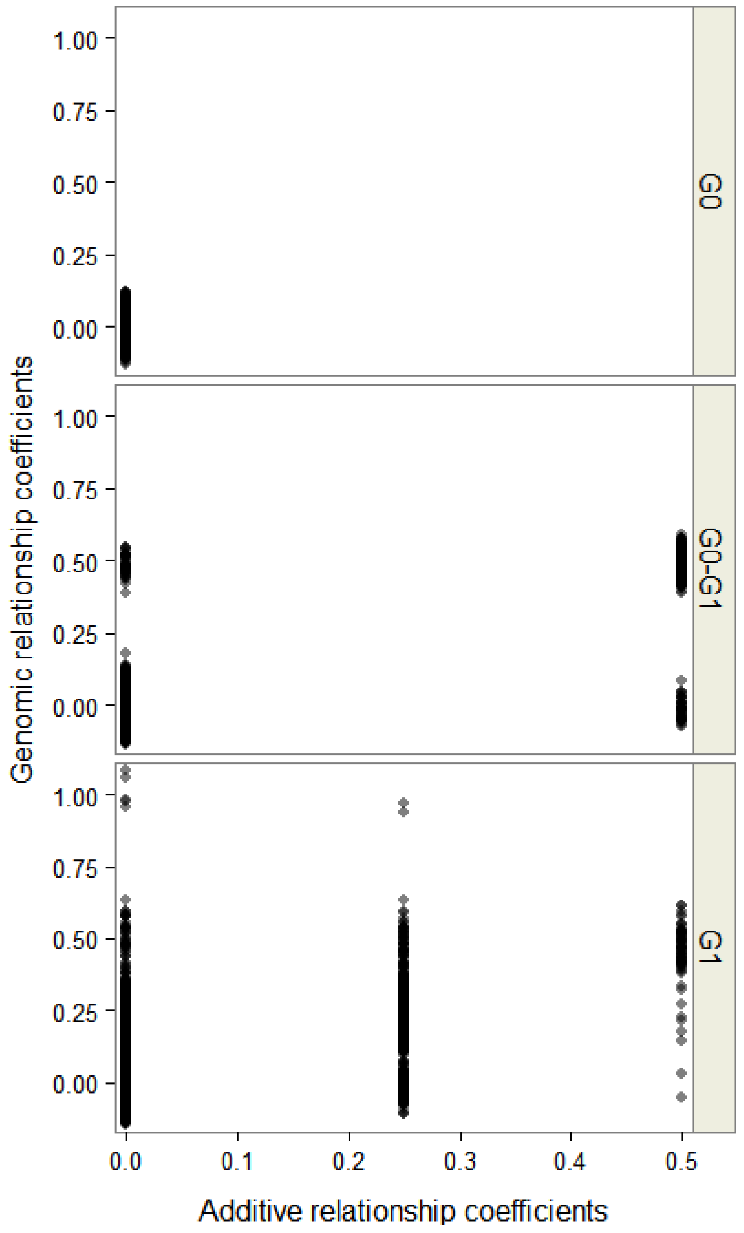

S7 Fig. Scatterplot of additive relationship coefficients and genomic relationship coefficients within generations (G0 or G1) and between generations (G0-G1). The 39 G1 and 9 G0 individuals presenting inconsistencies between pedigree-based and marker-based kinship were removed from the association analysis.
