# Supplementary material for: Linkage and Association Mapping for Two Major Traits Used in the Maritime Pine Breeding Program: Height Growth and Stem Straightness
Source: PLoS One. 2016 Nov 2;11(11):e0165323. doi: 10.1371/journal.pone.0165323 (PMC5091878; doi:10.1371/journal.pone.0165323)
Supplement: S8 Fig — The absolute values of markers are plotted on the 12 linkage groups of the Pinus pinaster composite map. (PDF) [file pone.0165323.s009.pdf]

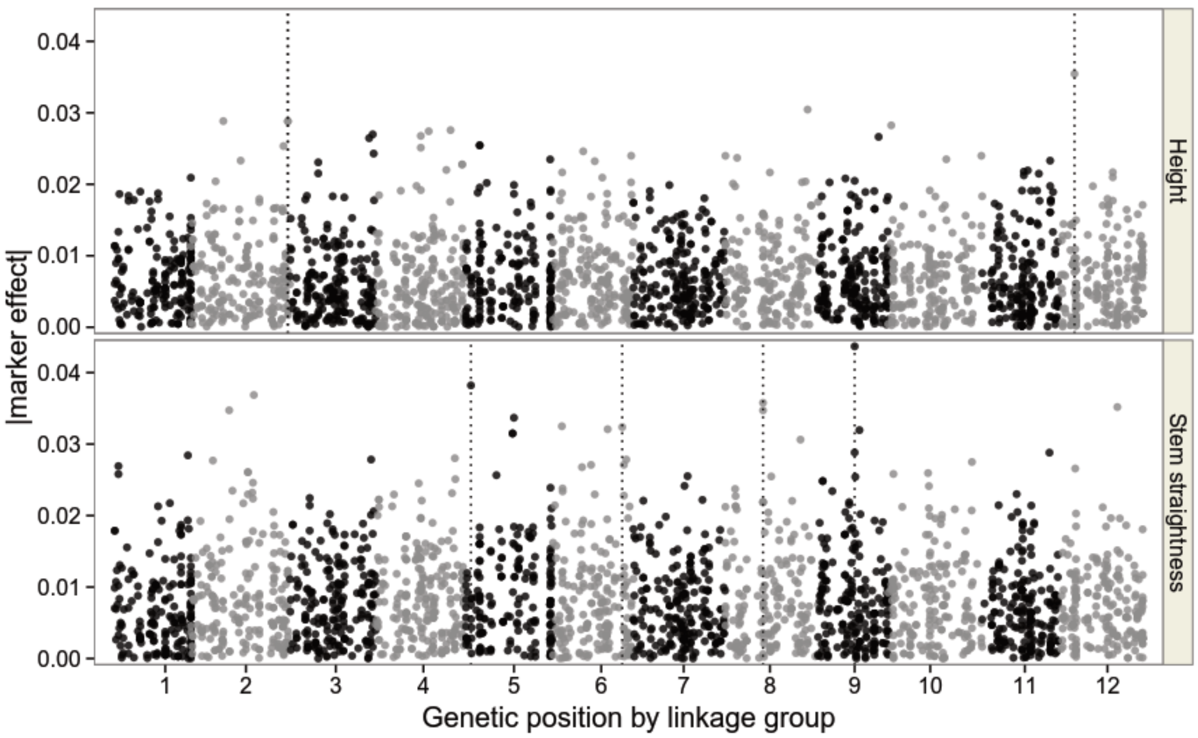

S8 Fig. Single marker effects on total height and stem straightness, in ridge regression BLUP (RR-BLUP). The absolute values of markers are plotted on the 12 linkage groups of the *Pinus pinaster* composite map.
