# Supplementary material for: Linkage and Association Mapping for Two Major Traits Used in the Maritime Pine Breeding Program: Height Growth and Stem Straightness
Source: PLoS One. 2016 Nov 2;11(11):e0165323. doi: 10.1371/journal.pone.0165323 (PMC5091878; doi:10.1371/journal.pone.0165323)
Supplement: S1 Note — (PDF) [file pone.0165323.s010.pdf]

S1 Note: Design of SNP arrays for the genotyping of the F2 mapping population

Two 384-SNP VeraCode arrays were constructed with SNPs derived from RNAseq data and from an already designed 1,536-GoldenGate SNP array [1]. The flowchart for the identification of informative SNPs/ INDELs in the H12 genotype (*i.e.* in the heterozygous state) is shown in S2 Fig. For the selection of SNPs derived from RNAseq data, we generated a composite cDNA library from total RNAs extracted from young and old needles, quiescent and swelling buds of genotype H12. We generated 172,892 reads with the Roche 454 Titanium platform. These sequences (available under accession SRX031594 at the NCBI short-read archive: http://www.ncbi.nlm.nih.gov/sra/SRX031594) were cleaned with the Pyrocleaner tool [2]. This step removed short (<150 bp) and long (>600 bp) reads, reads with more than 2% Ns (ambiguous base calls), low-complexity regions, and duplicated reads. We obtained a total of 96,851 cleaned reads, 79,179 of which were distributed between 9,241 contigs of the maritime pine PineContig_v2 unigene set [3]. We then used two Perl scripts from Lepoittevin *et al.* [4] for SNP detection: The first, *remove*, was used to remove positions containing alignment gaps for all reads. The second, *snp2illumina*, was used to extract SNPs and short INDELs of less than 7 bp in length, for output in a format compatible with the Illumina Assay Design Tool software (ADT, http://www.illumina.com). Finally, for each SNP, the minimum allele frequency (MAF), minimum allele number (MAN), depth and frequencies of each nucleotide were computed with the *SNP_statistics* script.

For the first 384-SNP array (Array 1 in S2 Fig.), the parameters used to identify SNPs from RNASeq data were as follows: depth $\geq$ 5, MAN > 1 and MAF $\geq$ 0.2 (Filter 1 in S2 Fig.). Only contigs with 1 or 2 SNPs separated by at least 60 bp were retained. In total, 404 SNPs were retained, including 106 INDELs. For the second 384-SNP array (Array 2), we used three different filters: Filter 2: depth $\geq$ 6, MAN $\geq$ 3 and MAF = 50% (corresponding to 49 SNPs), Filter 3: depth $\geq$ 7, MAN $\geq$ 3 and MAF ≥ 40 % (55 SNPs), and Filter 4: depth $\geq$8 (the most stringent in terms of coverage), MAN $\geq$ 3 and MAF $\geq$ 30% (198 SNPs). We kept contigs with only two SNPs per contig and an Illumina score ≥ 0.5. INDELs were excluded and SNPs in the first 60 bp of the contig were discarded. This approach led to the selection of 302 SNPs in total. The two arrays were completed with *in vitro* SNPs from Chancerel *et al.* [1]. We split a total of 179 *in vitro* SNPs segregating in the same F2 pedigree for which the genes had been localized on the H12 map between the two arrays, as follows: i) 97 SNPs in Array 1 and ii) 82 SNPs in Array 2. These SNPs were selected on the basis of linkage group coverage (even distribution) and ADT scores. We

used BlastN (Evalue=10^{-4}) analysis to avoid overlap between these 179 SNPs and *in silico* SNPs extracted from Pine_contigV2.

In addition to these two VeraCode arrays, 102 SNPs with known positions on the H12 map [3] were selected and included in four multiplexes, with MassArray design 4.1 software (Sequenom, SanDiego, CA, USA). Two multiplexes (with 22 and 12 SNPs) had already been developed by Chancerel *et al.* [3]. Two new multiplexes (with 36 and 32 SNPs) were designed for this study. The SNPs for these multiplexes were selected on the basis of their distribution on the genetic map, to improve map coverage.

1. Chancerel E, Lepoittevin C, Le Provost G, Lin Y-C, Jaramillo-Correa J, et al. (2011) Development and implementation of a highly-multiplexed SNP array for genetic mapping in maritime pine and comparative mapping with loblolly pine. BMC Genomics 12: 368.
2. Mariette J, Noirot C, Klopp C (2011) Assessment of replicate bias in 454 pyrosequencing and a multi-purpose read-filtering tool. BMC Research Notes 4: 149.
3. Chancerel E, Lamy J-B, Lesur I, Noirot C, Klopp C, et al. (2013) High-density linkage mapping in a pine tree reveals a genomic region associated with inbreeding depression and provides clues to the extent and distribution of meiotic recombination. BMC Biology 11: 50.
4. Lepoittevin C, Frigerio J-M, Garnier-Géré P, Salin F, Cervera M-T, et al. (2010) *In Vitro vs In Silico* Detected SNPs for the Development of a Genotyping Array: What Can We Learn from a Non-Model Species? PLoS ONE 5: e11034.
