# Supplementary material for: Linkage and Association Mapping for Two Major Traits Used in the Maritime Pine Breeding Program: Height Growth and Stem Straightness
Source: PLoS One. 2016 Nov 2;11(11):e0165323. doi: 10.1371/journal.pone.0165323 (PMC5091878; doi:10.1371/journal.pone.0165323)
Supplement: S2 Note — (PDF) [file pone.0165323.s011.pdf]

S2 Note: Genotyping of the F2 progeny

For the two Illumina arrays, 484 samples (grandparents C10 and L146, 5 replicates of the hybrid parent H12 to estimate reproducibility, and 477 F2s) were genotyped. Signal intensities were quantified with GenomeStudio (Genotyping module V1.9, Illumina, San Diego, USA). SNPs were individually inspected and classified as polymorphic if three clusters could clearly be identified (two homozygote classes and one heterozygote class), monomorphic if only one cluster was observed (one homozygote class), and as "failed" if the intensity was too low for interpretation or if cluster compression or abnormal clustering was observed.

For Assay #1, nine DNA samples were discarded because of the signal intensity was too low or due to location too far from the center of the cluster. For the remaining 468 samples, the mean call rate (percentage of SNPs successfully genotyped for each DNA sample) was 92%. The overall success rate (number of polymorphic SNPs divided by the total number of SNPs) was 32.8% (S4 Table). As expected, all previously mapped SNPs not classified as "failed" (i.e. 90%), were polymorphic. By contrast, 59% of the *in silico* SNPs were monomorphic, 13.5% were polymorphic and 27.5% "failed". We retrospectively checked the SNP depth parameters for each class, to identify the cause of this unexpectedly high frequency of monomorphic loci. On average, monomorphic SNPs presented six reads with a MAF of 0.25, failed SNPs presented seven reads with a MAF of 0.28, whereas polymorphic SNPs had nine reads with a MAF of 0.36. The rate of polymorphism (number of polymorphic SNPs divided by the number of successful SNPs) was therefore strongly dependent on SNP depth.

For Array #2, 27 DNA samples were discarded according to the same criteria used in Assay #1. For the 445 remaining samples, the mean call rate before analysis was 88%, reaching 99% after manual adjustment. This assay included 302 *in silico* SNPs and 82 validated SNPs. Most of the validated SNPs were informative in the mapping population (S4 Table): 68% of these SNPs were polymorphic, none was monomorphic as expected, and the assay failed for 32%. For *in silico* SNPs, 10% were monomorphic, 32% polymorphic and the assay failed for 58%. This failure rate is higher than the typical failure rate in pine for *in silico* SNPs (about 30%). We observed that the SNP failure rate reached 70% when two SNPs from the same contig were less than 60 bp apart. It is, therefore, very important to take this last criterion into account for SNP assay design with VeraCode technology. The reproducibility of this method was assessed with SNPs located on the same contig. We used a total of 26 contigs containing more than one SNP on the two

arrays. For the 11,989 genotype comparisons, we observed 34 recombination events, indicating an error rate of 0.3%.

In the two Sequenom assays developed in this study, 381 individuals were genotypes, together with two replicates of the parental H12 genotype. Negative controls were also included in the genotyping process, to detect contamination. We used a total of 15 ng of high-quality DNA for each reaction. Genotyping was carried out with the iPLEX Gold genotyping kit, in accordance with the manufacturer's instructions. Mass spectra were analyzed with MassArray Typer version 4.0.22 software, to generate genotype calls. All the SNPs were inspected by eye, to detect incorrect assignments by the "Autocluster" option of MassArray Typer. For the 36-plex, we found only two failed SNPs and for the 32-plex, the assay failed for six markers. Combining the two multiplex assays, we were able to genotype 60 SNPs correctly in 381 offspring, corresponding to a success rate of 88 %. Comparison of the two replicates of the H12 genotype revealed no genotyping inconsistencies, confirming the high degree of reproducibility of this method. For the four multiplexes (this study and [1]), 76 of the 102 genotyped SNPs were available for genetic mapping. A list of the 76 usable SNPs and associated primer pairs is provided in S5 Table.

1. Chancerel E, Lamy J-B, Lesur I, Noirot C, Klopp C, Ehrenmann F, et al. High-density linkage mapping in a pine tree reveals a genomic region associated with inbreeding depression and provides clues to the extent and distribution of meiotic recombination. BMC Biology. 2013;11(1):50. PubMed PMID: doi:10.1186/1741-7007-11-50.
