# Supplementary material for: Linkage and Association Mapping for Two Major Traits Used in the Maritime Pine Breeding Program: Height Growth and Stem Straightness
Source: PLoS One. 2016 Nov 2;11(11):e0165323. doi: 10.1371/journal.pone.0165323 (PMC5091878; doi:10.1371/journal.pone.0165323)
Supplement: S2 Table — For the F2 mapping population: stem straightness (STR) and height growth (HT) and for the G2 mapping population: annual height increment (HI) from 1985 to 1997. (PDF) [file pone.0165323.s013.pdf]

S2 Table Descriptive statistics for the traits measured in the F2 and G2 mapping populations. For the F2 mapping population: stem straightness (STR) and height growth (HT) and for the G2 mapping population: annual height increment (HI) from 1985 to 1997.

Mapping population	Trait	Mean	SD	CV	Min	Max	N
F2	HT	574.4	88	0.15	330	820	477
	STR	21.3	13.5	0.64	0	78	476
G2	HI_85-86	34.2	13.9	0.41	8	80	193
	HI_86-87	49.1	21.8	0.44	14	113	194
	HI_87-88	68.6	30.1	0.44	15	145	197
	HI_88-89	86.3	30.6	0.35	20	174	197
	HI_89-90	73	21.2	0.29	23	146	197
	HI_90-91	64.5	13.9	0.22	32	114	197
	HI_91-92	83.1	17.1	0.21	30	140	197
	HI_92-93	81.9	13.8	0.17	48	148	197
	HI_93-94	79.2	13.5	0.17	29	117	197
	HI_94-95	80.5	15.8	0.2	28	140	197
	HI_95-96	75.7	15.6	0.21	29	119	197
	HI_96-97	76.4	18.9	0.25	18	130	195
