# Supplementary material for: Linkage and Association Mapping for Two Major Traits Used in the Maritime Pine Breeding Program: Height Growth and Stem Straightness
Source: PLoS One. 2016 Nov 2;11(11):e0165323. doi: 10.1371/journal.pone.0165323 (PMC5091878; doi:10.1371/journal.pone.0165323)
Supplement: S3 Table — Only significant correlations (p < 0.05) are listed. (PDF) [file pone.0165323.s014.pdf]

S3 Table Spearman correlations for height increment in the G2 mapping population. Only significant correlations ($p < 0.05$) are listed.

	HI_85-86	HI_86-87	HI_87-88	HI_88-89	HI_89-90	HI_90-91	HI_91-92	HI_92-93	HI_93-94	HI_94-95	HI_95-96
HI_86-87	0.46										
HI_87-88	0.41	0.33									
HI_88-89	0.53	0.39	0.61								
HI_89-90	0.42	0.27	0.43	0.44							
HI_90-91	0.28	0.27	0.36	0.37	0.37						
HI_91-92	0.23	0.21	0.25	0.28	0.33	0.22					
HI_92-93	0.29	0.31	0.3	0.39	0.34	0.39	0.4				
HI_93-94	0.32	0.42	0.37	0.44	0.35	0.43	0.33	0.64			
HI_94-95	0.29	0.36	0.37	0.39	0.36	0.45	0.23	0.6	0.71		
HI_95-96	0.26	0.32	0.29	0.36	0.26	0.35	0.23	0.42	0.51	0.62	
HI_96-97	-	0.2	0.3	0.36	0.29	0.36	0.24	0.41	0.48	0.55	0.56
