# Supplementary material for: Linkage and Association Mapping for Two Major Traits Used in the Maritime Pine Breeding Program: Height Growth and Stem Straightness
Source: PLoS One. 2016 Nov 2;11(11):e0165323. doi: 10.1371/journal.pone.0165323 (PMC5091878; doi:10.1371/journal.pone.0165323)
Supplement: S4 Table — (PDF) [file pone.0165323.s015.pdf]

S4 Table Statistics for the two VeraCode SNP arrays

		Number of SNPs	ADT score	Number of SNPs		
				Polymorphic	Monomorphic	Failed
Array 1	Already mapped SNP	97	0.89 [0.68–1]	87 (90%)	0	10 (10%)
	In silico SNP	287	0.79 [0.65–0.99]	39 (13.5%)	169 (59%)	79 (27.5%)
	Total	384	0.82 [0.65–1]	126 (32.8%)	169 (44%)	89 (23.2%)
Array 2	Already mapped SNP	82	0.8 [0.5–0.985]	56 (68%)	0	26 (32%)
	In silico SNP	302	0.73 [0.5–0.99]	97 (32%)	30 (10%)	175 (58%)
	Total	384	0.74 [0.5–0.99]	153 (39.8%)	30 (7.8%)	201 (52.3%)
