# Supplementary material for: Linkage and Association Mapping for Two Major Traits Used in the Maritime Pine Breeding Program: Height Growth and Stem Straightness
Source: PLoS One. 2016 Nov 2;11(11):e0165323. doi: 10.1371/journal.pone.0165323 (PMC5091878; doi:10.1371/journal.pone.0165323)
Supplement: S6 Table — (PDF) [file pone.0165323.s017.pdf]

S6 Table Current status of major international conifer genome sequencing projects.

Species	Genome size	Type of material used for sequencing	Sequencing and assembly strategy	Number of scaffolds & N50	Number of gene models predicted
Pinus pinaster	51-60 pg / 2C<br>[1]	DNA from a single haploid line generated by *in vitro* culture of a megagametophyte	Short reads sequencing (Illumina) + mate pair sequencing 454.<br>Assembly: MaSuRCA + SOAPdenovo2.	Scaffolds >500 bp: 4.4 millions<br>N50: ~920 bp<br>Assembly version 0.8.	-
Picea abies	40 pg / 2C<br>[2]	DNA from haploid and diploid tissues of the same genotype	Haploid WGS combined with diploid fosmid pools and diploid WGS. Short reads sequencing (Illumina)<br>Assembly: GAM-NGS, BESST	Scaffolds >500 bp: 10.2 millions<br>N50: ~600bp<br>Assembly version 1.0	28,354
Pinus taeda	44 pg / 2C<br>[3,4]	DNA from haploid and filtered diploid tissue of the same genotype.	Haploid WGS combined with filtered diploid WGS + short read sequencing PE and MP (Illumina)<br>Assembly: MaSuRCA	Scaffolds >500bp: 2.2 millions<br>N50: 76 Kbp<br>Assembly version 1.01	9,024 (full-length)<br>32,432 (partial/ low quality
Picea glauca	42 Gb / 2C<br>[5,6]	DNA from diploid tissues	WGS + short read sequencing PE and MP (Illumina)<br>Assembly: ABySS	Scaffolds >500bp: 4.9 millions<br>N50: 20.4 Kbp	23,589 (full-length)
Pinus sylvestris	50-54pg / 2C	DNA from haploid and diploid tissues from the same individual	Haploid combined with diploid WGS Short read sequencing (Illumina)<br>Assembly: CLC	Scaffolds >500bp: 6.5 millions<br>N50: 2245 bp	-
Pinus sibirica	60 pg / 2C<br>[7]	DNA from haploid megagametophytes and diploid needle tissues of the same genotype	Short read sequencing PE and MP 2× 100 sequencing (Illumina)<br>Assembly: CLC Assembly Cell, ABySS and MaSuRCA	Scaffolds: in progress<br>Contigs >500 bp: 9.9 millions<br>N50: ~3200 bp<br>Assembly version 0.9	-

1. Chagné D, Lalanne C, Madur D, Kumar S, Frigério J-M, et al. (2002) A high density genetic map of maritime pine based on AFLPs. Ann For Sci 59: 627-636.
2. Nystedt B, Street NR, Wetterbom A, Zuccolo A, Lin Y-C, et al. (2013) The Norway spruce genome sequence and conifer genome evolution. Nature 497: 579-584.
3. Wegrzyn JL, Liechty JD, Stevens KA, Wu L-S, Loopstra CA, et al. (2014) Unique Features of the Loblolly Pine (*Pinus taeda* L.) Megagenome Revealed Through Sequence Annotation. Genetics 196: 891-909.
4. Neale DB, Wegrzyn JL, Stevens KA, Zimin AV, Puiu D, et al. (2014) Decoding the massive genome of loblolly pine using haploid DNA and novel assembly strategies. Genome Biology 15: 1-13.
5. Warren RL, Keeling CI, Yuen MMS, Raymond A, Taylor GA, et al. (2015) Improved white spruce (Picea glauca) genome assemblies and annotation of large gene families of conifer terpenoid and phenolic defense metabolism. The Plant Journal 83: 189-212.
6. Birol I, Raymond A, Jackman SD, Pleasance S, Coope R, et al. (2013) Assembling the 20 Gb white spruce (Picea glauca) genome from whole-genome shotgun sequencing data. Bioinformatics 29.
7. Zonneveld BJM (2012) Conifer genome sizes of 172 species, covering 64 of 67 genera, range from 8 to 72 picogram. Nordic Journal of Botany 30: 490-502.
